# Vibrational Spectrum of Magnesium Monochalcogenide Nanoparticles

**DOI:** 10.3390/nano14231918

**Published:** 2024-11-28

**Authors:** Nikos Aravantinos-Zafiris, Fotios I. Michos, Michail M. Sigalas

**Affiliations:** 1 Department of Environment, Ionian University, 29100 Zakynthos, Greece; 2 Department of Materials Science, University of Patras, 26504 Patras, Greece; fotismixos@gmail.com (F.I.M.); sigalas@upatras.gr (M.M.S.)

**Keywords:** vibration spectrum, nanoparticles, density functional theory, magnesium monochalcogenides

## Abstract

In this work, the vibrational spectra of magnesium monochalcogenide nanoparticles were examined numerically. The calculations were performed with Density Functional Theory and the examined magnesium monochalcogenide nanoparticles were formed from an initial cubic-like unit with type ${Mg}_{4}Y_{4}$, where Y=S,Se,Te, after elongating this unit along one, two, and three vertical directions. Therefore, beyond the initial building block, different groups of magnesium monochalcogenide nanoparticles were examined in the form ${Mg}_{x}Y_{x}$, where x=8,16,24. Especially for the case where the chalcogen part of the nanoparticle was sulfur, another group of nanoparticles was examined where x=32. For this group of the examined nanostructures, an exotic case was also included in the calculations. Among the findings of this research was the existence of stable structures, of the examined morphologies. The calculations of this research led to the identification of both common characteristics and differences among these nanostructures. These characteristics regarding their vibrational modes could be a very useful tool, especially for experimentalists. The relevant phonon spectrum that was extracted from the calculations also provided very useful information regarding the examined nanoparticles and their potential uses in several technological applications.

## 1. Introduction

The control of phonons, which are the physical particles that represent mechanical vibrations and cover a wide range of frequencies such as sound and heat transmissions, has gained over the last decades both scientific and technological attention [1]. At the nanoscale, phonons cover a wide frequency range from ultrasounds (MHz) to heat (THz), and thus, the manipulation of such phonon frequencies could result in several possible applications, therefore inducing an intense research interest and a relevant challenging perspective in the field [2]. Vibrational modes that are present in all nanoscale structures offer an exceptional source of information regarding their mechanical properties, which is a very useful tool for understanding a plethora of physical phenomena such as their interaction with other nanoparticles or their environment [3]. It should also be noted that the recent progress made with a deeper understanding of nanoscale solid-state systems, in accordance with experimental techniques and fabrication methods, has led to significant development in the field of nanophononics [4,5].

Semiconductor nanoparticles (NPs) have plenty of size-dependent properties [6,7]. Hybrid nanoparticulate systems that consist of organic hosts and can encapsulate inorganic NPs have attracted plenty of academic interest over the last years due to their several potential applications in several fields such as drug delivery and bioimaging [8]. The encapsulation of inorganic nanoparticles in supramolecular cavities was recently found to significantly modify the photophysics of the NPs [9]. When inorganic NPs such as carbon nanotubes, TiO_2_ NPs, and carbon quantum dots (QDs) are incorporated into polymethyl methacrylate matrices, they result in nanocomposite films with improved structural, morphological, and optical properties [10]. Nanoparticles at nanometer size vibrate within the frequency range between GHz and THz, and such motions for metal and semiconductor nanostructures were studied using time-resolved spectroscopy [11]. Nanoparticles of several sizes with magnesium were investigated with plenty of methods. The properties of nanoclusters with magnesium were studied by Jellinek and Acioli with the use of gradient-corrected DFT [12]. Recently, an ab initio study, which was performed for the examination of the properties of magnesium sulfide (MgS) and magnesium selenide (MgSe) semiconductors after the substitution by transition metals, showed that, except in the case of Mn, the rest of the examined structures exhibited a half-metallic ferromagnetic characteristic [13]. The electronic, mechanical, and thermal properties of magnesium-based chalcogenide buckled monolayers have been theoretically examined by using density functional theory for potential uses at the nanoscale [14].

Chalcogenide QDs have one or more of the chalcogen elements (sulfur (S), selenium (Se), and tellurium (Te)) as a constitutive component, and can be synthesized by a variety of techniques, and due to their structural and optical properties provide plenty of applications [15]. Varshney et al. have studied theoretically and predicted accurately the structural aspects induced by pressure from different types of magnesium chalcogenides [16]. The structural phase stability of the three magnesium chalcogenides MgTe, MgS, and MgSe was investigated within the frame of density functional theory by Gökoğlu et al. [17]. The combination of low cost together with high performance of QD Sensitized Solar Cells based on cadmium chalcogenide has also attracted scientific and technological interest [18]. Due to their low cost and because they are materials friendly for the environment with tunable morphologies, iron (Fe) chalcogenide NPs have been studied for a plethora of potential applications [19]. Tin (Sn)-based chalcogenide NPs have also been extensively studied for possible applications in photovoltaics [20]. The advances regarding the composition of groups IV–VI π-phase monochalcogenides led to nanoparticles for photodetecting and solar cell applications [21]. Copper-antimony and copper-bismuth chalcogenide NPs, due to their rich range of compounds, are suitable for solar cells and photovoltaics [22]. Recently, it was theoretically shown by Karantagli et al. that group IV monochalcogenide NPs provide exotic novel morphologies [23]. Wobbe and Zwijnenburg studied by using Density Functional Theory (DFT) and Time-Dependent DFT (TD-DFT) the optical gap for ${\left(MgX\right)}_{32}$ NPs where X=O,S,Se [24]. Chronis et al. have recently studied the properties of magnesium and aluminum hydride NPs by using the same numerical methods [25]. Furthermore, again by using DFT, TD-DFT, and Real-Time TD-DFT, Chronis et al. studied the properties of rods and disks at the nanoscale of beryllium, magnesium, and calcium [26].

The present study includes the numerical investigation of the IR Vibration Spectrum (VS) of magnesium monochalcogenide NPs with the use of DFT. The investigated structures are described by the general type ${Mg}_{x}Y_{x}$ where Y=S,Se,Te. The examined magnesium monochalcogenide nanoparticles were composed of an initial cubic-like building block of the form ${Mg}_{4}Y_{4}$, where Y=S,Se,Te. The studied nanostructures were created after elongation along one, two, and three vertical directions of the initial building unit, which had a cubic shape. Aravantinos-Zafiris et al. have already studied, by using DFT and TD-DFT, the structural and optical properties of such NPs [27]. This investigation aims to supply numerical evidence, which can be useful for experimentalists, regarding the vibrations for a wide group of NPs. The findings of this work could become a powerful tool regarding the response of the examined NPs when interacting with electrons, magnons, or photons.

## 2. Computational Method

The most stable crystal structure of MgS, MgSe, and MgTe is the rocksalt structure, and from that structure, we derived the basic cube (${Mg}_{4}Y_{4}$) that was used to create all the nanoparticles examined in this study. For this study, DFT was used for the calculation of the vibrational spectrum of the examined NPs. First, the stability of the structure of the proposed NPs was investigated. The morphology of each NP that was examined was created by properly elongating the initial cubic nanostructure along one, two, and three vertical directions. The geometries were optimized by using the gradient-corrected functional of Perdew, Burke, and Ernzerhof (PBE) [28], and utilization of the triple-ζ quality def2-TZVP basis set [29]. The TURBOMOLE [30] package was used at all stages of this research. As already mentioned, the calculated ultraviolet–visible absorption spectra were published in a previous study by Aravantinos-Zafiris et al. [27]. Here, the vibrational spectra originated with the use of a script that represents each vibration as a Gaussian function, which has its center on the vibrational frequency considering a standard deviation (broadening) of 10 cm^−1^. This broadening reflects the resolution of the calculated spectra and aligns with the typical accuracy of the PBE functional combined with the def2-TZVP basis set, which provides vibrational frequencies with an error bar of approximately ±10–15 cm⁻¹ when compared to experimental data. A representative example, where all the peaks of the vibrational spectra are collected in a table, was included as a Supplementary Materials that presents the vibrational peaks for the ${Mg}_{24}S_{24}$ structures. It is important to mention at this point that additional calculations were already performed with the Particle Swarm Optimization (PSO) to ensure that the studied NPs are indeed realistic [27]. These calculations, which are computationally costly and time-consuming, were limited to ${Mg}_{4}Y_{4}$ and ${Mg}_{8}Y_{8}$ and validated the stability of the studied nanostructures while also classifying them in the midst of the best of the considered within the framework of PSO calculations.

## 3. Results

### 3.1. Mg_x_S_x_ Cases

**Figure 1:**
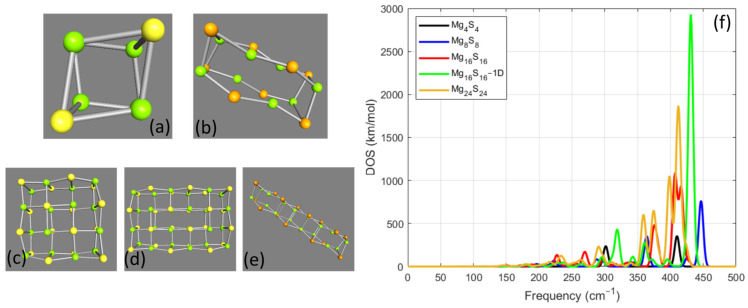
The geometry optimized ${Mg}_{x}S_{x}$ nanoparticles for x≤24. The optimized geometry for (**a**) ${Mg}_{4}S_{4}$, (**b**) ${Mg}_{8}S_{8}$, (**c**) ${Mg}_{16}S_{16}$, (**d**) ${Mg}_{24}S_{24}$, and (**e**) ${Mg}_{16}S_{16}-1D$. (**f**) The vibrational spectrum for the ${Mg}_{x}S_{x}$ nanoparticles for x≤24. Figure legend indicates the color that represents each line of the plot.

The first examined group of NPs was the ${Mg}_{x}S_{x}$. The group of structures that were studied in this Section is presented in Figure 1. In more detail, the structures after the geometry optimization are collected at the right part of Figure 1, and the relevant VS for all the examined cases is shown on the right side in Figure 1f. The optimized geometry for the ${Mg}_{4}S_{4}$ case is shown in Figure 1a, and the relevant VS is presented with the black line in Figure 1f. For this NP, the calculations provided two resonance frequencies where the first was at 301 ${cm}^{-1}$ with DOS value 234 km/mol and the second at 410 ${cm}^{-1}$ with DOS value 356 km/mol. It should be also mentioned that there is another weak resonance with frequency 205 ${cm}^{-1}$ and relevant DOS value 3.4 km/mol. The optimized geometry for ${Mg}_{8}S_{8}$ case is shown in Figure 1b. For ${Mg}_{8}S_{8}$, there is a first weak resonance at 174 ${cm}^{-1}$ with 7 km/mol as can be seen from the relevant plot, which is indicated with the blue line in Figure 1f. Another two resonances appear at 196 ${cm}^{-1}$ with DOS value 29 km/mol and at 222 ${cm}^{-1}$ with DOS value 66 km/mol. There is also another resonance at 251 ${cm}^{-1}$ with 11 km/mol. Another close couple of resonances were calculated at frequencies 288 ${cm}^{-1}$ and 297 ${cm}^{-1}$ with DOS values 84 km/mol and 59 km/mol, respectively. In the case of ${Mg}_{8}S_{8}$, two stronger resonances were calculated at frequencies 364 ${cm}^{-1}$ and 447 ${cm}^{-1}$. The DOS values for these cases were found 354 km/mol and 761 km/mol, respectively. Very close to each of these resonances were found two weaker resonances at 345 ${cm}^{-1}$ with 48 km/mol and at 426 with 110 km/mol.

The next case of the ${Mg}_{x}S_{x}$ NPs that were studied was ${Mg}_{16}S_{16}-1D$, where the sign 1D indicates the elongation of the studied NP along one direction, as can be seen in Figure 1e, where the shape of the structure after the geometry optimization is presented. The VS for this case is shown with the green line in Figure 1f. As can be seen from the plot, there was a strong resonance that was found at 431 ${cm}^{-1}$ with DOS value 2930 km/mol. Another two notable resonances were found at 319 ${cm}^{-1}$ and at 361 ${cm}^{-1}$ with DOS values 434 km/mol and 280 km/mol, respectively. Three resonances were calculated at frequencies 295 ${cm}^{-1}$, 343 ${cm}^{-1}$, and 396 ${cm}^{-1}$ with relevant DOS values 107 km/mol, 114 km/mol, and 84 km/mol, respectively. In addition, there were also some weaker resonances that were found at lower frequencies. In more detail, resonance frequencies were also found at 179 ${cm}^{-1}$ with 26 km/mol, at 201 ${cm}^{-1}$ with 18 km/mol, at 227 ${cm}^{-1}$ with 41 km/mol, and at 264 ${cm}^{-1}$ with 32 km/mol. The case of ${Mg}_{16}S_{16}$ NP was examined next. This structure was built after elongating the cubic ${Mg}_{4}S_{4}$ along the two perpendicular directions. The shape of this NP after geometry optimization is shown in Figure 1c. The DOS plot for this case is presented with the red line in Figure 1f. For this case, two strong resonance frequencies were found at 407 ${cm}^{-1}$ with 1088 km/mol and at 416 ${cm}^{-1}$ with 936 km/mol. Another strong resonance for this case was calculated at 376 ${cm}^{-1}$ with DOS value 482 km/mol. Weaker resonances were also found at lower frequencies. In more detail, these resonances were found at 227 ${cm}^{-1}$ with 135 km/mol, at 270 ${cm}^{-1}$ with 170 km/mol, at 298 ${cm}^{-1}$ with 79 km/mol, at 316 ${cm}^{-1}$ with 30 km/mol, and at 339 ${cm}^{-1}$ with 53 km/mol. Four more weak resonances were also calculated at a lower frequency range. These resonances were found at 150 ${cm}^{-1}$, 171 ${cm}^{-1}$, 189 ${cm}^{-1}$, and 207 ${cm}^{-1}$. For these cases, the DOS value does not exceed 24 km/mol.

The study continued with the case of ${Mg}_{24}S_{24}$. The optimized shape of this NP is shown in Figure 1d, and the DOS spectrum is represented by the orange line in Figure 1f. For this examined case, there was a strong vibrational mode for frequency 412 ${cm}^{-1}$ with 1869 km/mol. Close to this mode, there was another one with frequency 399 ${cm}^{-1}$ with 1046 km/mol. Another couple of resonances were found for frequencies 359 ${cm}^{-1}$ and 374 ${cm}^{-1}$ with relevant DOS values 605 km/mol and 650 km/mol, respectively. At lower frequencies, there were also some frequencies found at 233 ${cm}^{-1}$ with 124 km/mol, at 263 ${cm}^{-1}$ with 78 km/mol, and at 291 ${cm}^{-1}$ with 231 km/mol. It should be also noted that weaker resonances were found at lower frequencies such as a resonance at 145 ${cm}^{-1}$ with 13.8 km/mol, at 182 ${cm}^{-1}$ with 27.7 km/mol, and at 216 ${cm}^{-1}$ with 54 km/mol. Another triplet of weak resonances was found at frequencies 311 ${cm}^{-1}$ with 51 km/mol, at 324 ${cm}^{-1}$ with 22 km/mol, and at 340 ${cm}^{-1}$ with 40 km/mol.

#### Mg_32_S_32_ Cases

**Figure 2:**
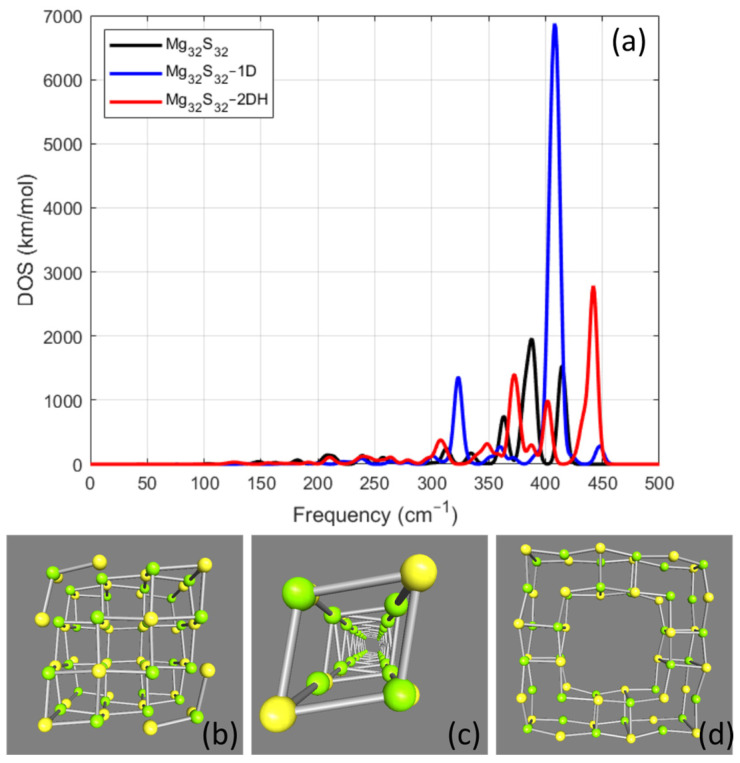
(**a**) The vibrational spectrum for the ${Mg}_{32}S_{32}$ cases of nanoparticles. Figure legend indicates the color that represents each line of the plot. Bottom panel presents the geometry-optimized cases of ${Mg}_{32}S_{32}$. The optimized geometry for (**b**) ${Mg}_{32}S_{32}$, (**c**) ${Mg}_{32}S_{32}-1D$, and (**d**) ${Mg}_{32}S_{32}-2DH$.

The next case of the ${Mg}_{x}S_{x}$ group of the examined NPs was ${Mg}_{32}S_{32}$. For this case, three different geometries were examined, and the relevant results of each VS are collected in the plot on the top panel in Figure 2. Relevantly the morphology of each structure after geometry optimization is shown on the bottom panel in Figure 2. The first case of this group of NPs is the cubic ${Mg}_{32}S_{32}$ where the NP was constructed after elongating the cubic ${Mg}_{4}S_{4}$ one time along the three perpendicular directions. The shape of this structure after the geometry optimization is shown in Figure 2b, and the relevant DOS spectrum is represented by the black line in Figure 2a. For this case, the DOS spectrum shows three notable resonances at frequencies 363 ${cm}^{-1}$, 388 ${cm}^{-1}$, and 415 ${cm}^{-1}$ with DOS values 745 km/mol, 1959 km/mol, and 1527 km/mol, respectively. Another couple of frequency modes were calculated at 313 ${cm}^{-1}$ with 246 km/mol and 335 ${cm}^{-1}$ with 172 km/mol. Another group of resonances was found within the frequency range between 140 ${cm}^{-1}$ and 280 ${cm}^{-1}$ with DOS values less than 140 km/mol. The second examined case of this group of NPs was the one in which the examined nanostructure was created after elongating the cubic unit block along one direction, symbolized as ${Mg}_{32}S_{32}-1D$. The shape of this NP after the geometry optimization is shown in Figure 2c. For this case, as shown with the blue line of the relevant plot in Figure 2a, only two notable resonance modes were calculated. The first at 323 ${cm}^{-1}$ with 1358 km/mol and the second at 408 ${cm}^{-1}$ with 6874 km/mol. The third case, which should be considered as an exotic NP [27], was made by elongation of the initial cubic unit cell two times along the two perpendicular directions, thus creating a ${Mg}_{36}S_{36}$ NP. The exotic ${Mg}_{32}S_{32}$ NP was created from the ${Mg}_{36}S_{36}$ when a cubic unit ${Mg}_{4}S_{4}$ cell was removed properly from the center, thus forming a hole in the center of the NP, as shown in Figure 2d. Aravantinos-Zafiris and Sigalas have already numerically examined cases where defects in different types of systems at the nanoscale could enrich them with vibrational properties such as phononic band gaps [31] or make the potential candidates for applications relevant to phononic interconnects [32]. Therefore, for the nanoparticles related to this research, this exotic case is symbolized as ${Mg}_{36}S_{36}-2DH,$ indicating the elongation along the two directions and the creation of the hole in the NP. Thus, for the ${Mg}_{36}S_{36}-2DH$ NP, three strong resonances at frequencies 373 ${cm}^{-1}$, 402 ${cm}^{-1}$, and 442 ${cm}^{-1}$ with DOS values 1394 km/mol, 994 km/mol, and 2782 km/mol, respectively.

### 3.2. Mg_x_Se_x_ Cases

**Figure 3:**
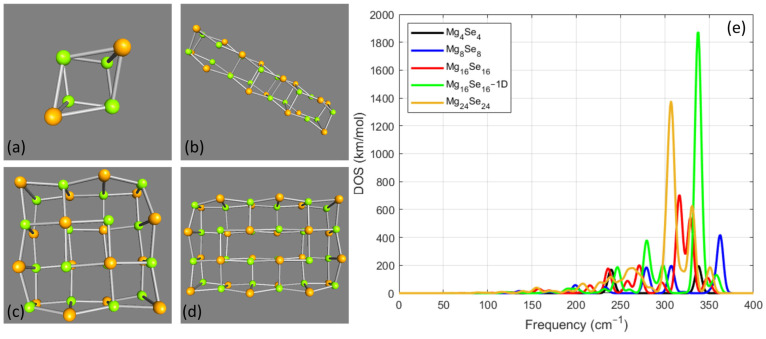
Selected cases of the geometry-optimized ${Mg}_{x}{Se}_{x}$ nanoparticles for x≤24. The optimized geometry for (**a**) ${Mg}_{4}{Se}_{4}$, (**b**) ${Mg}_{16}{Se}_{16}-1D$, (**c**) ${Mg}_{16}{Se}_{16}$, (**d**) ${Mg}_{24}{Se}_{24}$. (**e**) The vibrational spectrum for the ${Mg}_{x}{Se}_{x}$ nanoparticles for x≤24. Figure legend indicates the color that represents each line of the plot.

The next group of the investigated magnesium chalcogenide NPs was composed of selenium (Se) as the chalcogenide part of the NP. The results for this set of NPs were collected in Figure 3. In the left part of the figure, selected cases of the examined NPs are presented after the geometry optimization of each one. On the left side of Figure 3, the VS of each case is collected. For the simplest case of ${Mg}_{4}{Se}_{4}$, the optimized structure is shown in Figure 1a, and the relevant DOS spectrum is represented by the black line in Figure 3e. The calculations provided two resonance frequencies where the first was at 239 ${cm}^{-1}$ with DOS value 169 km/mol and the second at 338 ${cm}^{-1}$ with DOS value 196 km/mol. As expected, more resonances are present for the case of ${Mg}_{8}{Se}_{8}$. In more detail, as shown with the blue line of the plot in Figure 3e, there is a resonant peak at 362 ${cm}^{-1}$ with DOS value 417 km/mol, and two other resonances at 307 ${cm}^{-1}$ with 197 km/mol and at 279 ${cm}^{-1}$ with 186 km/mol. At lower frequencies, there are also frequencies where the NP is excited such as 233 ${cm}^{-1}$ and 199 ${cm}^{-1}$ with DOS values 44 km/mol and 58 km/mol, respectively. The elongation of the initial cubic unit cell along one direction has led to the one-dimensional case of ${Mg}_{16}{Se}_{16}$, indicated as ${Mg}_{16}{Se}_{16}-1D$, as shown in Figure 3b. For this case, as can be seen from the green line of the plot in Figure 3e, a strong resonance was found at 338 ${cm}^{-1}$ with 1872 km/mol. Close to this mode another mode was found at 358 ${cm}^{-1}$ with 131 km/mol. Three more resonances were also calculated for this case at 298 ${cm}^{-1}$ with 201 km/mol, at 280 ${cm}^{-1}$ with 378 km/mol, and at 246 ${cm}^{-1}$ with 187 km/mol. The DOS spectrum for the relevant case of ${Mg}_{16}{Se}_{16}$ where the examined NP was built after elongating the initial cubic unit cell along the two vertical directions, as shown in Figure 3c, is presented by the red line in Figure 1e. As can be seen from the diagram, more resonance frequencies appear for this case compared to the one-dimensional ${Mg}_{16}{Se}_{16}-1D$. The strongest pair of resonances appears at 316 ${cm}^{-1}$ with 702 km/mol and at 329 ${cm}^{-1}$ with 544 km/mol. Notable mode frequencies were also calculated at 236 ${cm}^{-1}$ with 178 km/mol and at 271 ${cm}^{-1}$ with 201 km/mol. The highest value of the resonance frequency was found at 348 ${cm}^{-1}$ with 112 km/mol. For the group of ${Mg}_{x}{Se}_{x}$ NPs, the study concluded with the case ${Mg}_{24}{Se}_{24}$, which is shown in Figure 3d. The relevant DOS spectrum is shown with the orange line of the plot of Figure 3e. The strongest resonance for this case was calculated for frequency 307 ${cm}^{-1}$ with 1378 km/mol. Another strong resonance was calculated at 331 ${cm}^{-1}$ with 629 km/mol. For the ${Mg}_{24}{Se}_{24}$ NP, the highest value of the resonance frequency was found at 351 ${cm}^{-1}$ with 187 km/mol. Another notable point for this case is that there is a frequency zone between 200 and 280 ${cm}^{-1}$ where the frequency resonances are very close, thus resulting in a kind of continuum of the DOS spectrum where the maximum value is 180 km/mol.

### 3.3. Mg_x_Te_x_ Cases

**Figure 4:**
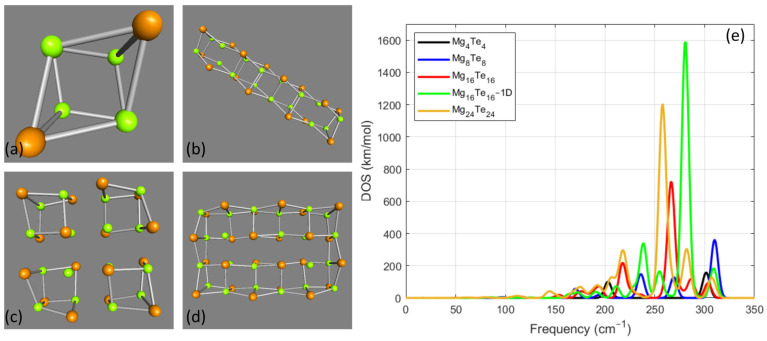
Selected cases of the geometry-optimized ${Mg}_{x}{Te}_{x}$ nanoparticles for x≤24. The optimized geometry for (**a**) ${Mg}_{4}{Te}_{4}$, (**b**) ${Mg}_{16}{Te}_{16}-1D$, (**c**) ${Mg}_{16}{Te}_{16}$, (**d**) ${Mg}_{24}{Te}_{24}$. (**e**) The vibrational spectrum for the ${Mg}_{x}{Te}_{x}$ nanoparticles for x≤24. Figure legend indicates the color that represents each line of the plot.

The last group of magnesium chalcogenide NPs that was examined was the one that was composed of tellurium (Te) as the chalcogenide atoms of the NP. The results of this part regarding the VS, in combination with the selected studied nanostructures, are collected in Figure 4. As in the previous examined cases, for the simplest case of ${Mg}_{4}{Te}_{4}$, which is shown in Figure 4a, the calculations provided two resonance frequencies as can be seen from the black line in the DOS spectrum in Figure 4e. The first was at 203 ${cm}^{-1}$ with DOS value 101 km/mol, and the second at 301 ${cm}^{-1}$ with DOS value 157 km/mol. The case of ${Mg}_{8}{Te}_{8}$ provided five resonances that appear in the DOS spectrum, which is represented by the blue line of the plot. The strongest resonance is at 310 ${cm}^{-1}$ with 362 km/mol. There are also four resonant peaks at 170 ${cm}^{-1}$ with DOS value 57 km/mol, at 195 ${cm}^{-1}$ with 20 km/mol, at 236 ${cm}^{-1}$ with 149 km/mol, and at 269 ${cm}^{-1}$ with 129 km/mol. As in the previously examined magnesium chalcogenide NPs, the elongation of the initial cubic cell four times along one direction led to the ${Mg}_{16}{Te}_{16}-1D$ NP. This case is presented in Figure 4b, and the relevant DOS spectrum is plotted with the green line. The strongest resonance for this case was at 281 ${cm}^{-1}$ with DOS value 1590 km/mol. The highest value of the resonance frequency for this NP was found at 309 ${cm}^{-1}$ with 185 km/mol. There are also three more notable resonances at 211 ${cm}^{-1}$, at 238 ${cm}^{-1}$, and at 255 ${cm}^{-1}$ with DOS values 72 km/mol, 340 km/mol, and 165 km/mol, respectively. The DOS spectrum for the case of ${Mg}_{16}{Te}_{16}$ where, as shown in Figure 1c, the examined NP was built after elongating the initial cubic unit cell along the two vertical directions is presented by the red line of Figure 1e. For this case, the highest frequency resonance was found at 303 ${cm}^{-1}$ with 91 km/mol. The strongest resonance for this case was found at 266 ${cm}^{-1}$ with 721 km/mol. Other notable resonance frequencies were found at 286 ${cm}^{-1}$ with an intensity of 118 km/mol and at 218 ${cm}^{-1}$ with an intensity of 218 km/mol. As in the relevant case for selenium NPs, this part concluded with the case ${Mg}_{24}{Te}_{24}$. For this case, where the geometry optimized structure is shown in Figure 4d, the strongest resonance was found at 258 ${cm}^{-1}$ with DOS value 1205 km/mol, and the highest value of the resonance frequency was 307 ${cm}^{-1}$ with 127 km/mol. There was also another notable resonance at 218 ${cm}^{-1}$ with DOS value 298 km/mol.

## 4. Discussion

The study of the vibrational modes of magnesium monochalcogenide NPs provided very useful content related to both their structural morphology and their constitutive counterparts. Moreover, the study enlightened some common properties of the NPs related to the number of atoms in the nanostructure and the selected constitutive chalcogenide element. The differences observed in the vibrational modes arise primarily from variations in the number of atoms and the geometrical configurations of the nanoparticles, rather than differences in their crystal structure. For example, the 1D structure in Figure 2 exhibits resonances with the strongest density of states (DOS) due to its distinct geometry compared to other configurations with the same number of atoms. It is expected that for all examined cases of ${Mg}_{x}Y_{x}$ NPs, as x increases, the number of the vibrational modes will also increase. It should be also mentioned that for all examined cases, the calculated vibrational spectrum did not provide negative frequencies for any of the nanostructures, therefore validating their structural stability. According to the studied nanostructures, it was considered more suitable to start with the observations related to the calculations of the NPs where x≤24. As will be analyzed, these cases constitute a group of NPs with similar characteristics and useful information can be extracted from the results of this set of calculations.

Among the cases where x≤24, the highest resonant frequency was achieved for x=8 for all chalcogenides. In more detail, the highest resonance frequency for ${Mg}_{8}S_{8}$ was 447 ${cm}^{-1}$ with DOS value 761 km/mol. For ${Mg}_{8}{Se}_{8}$, the highest resonance was 362 ${cm}^{-1}$ with DOS value 417 km/mol, and for ${Mg}_{8}{Te}_{8},$ it was 310 ${cm}^{-1}$ with DOS value 362 km/mol. For all these cases where x=8, this highest resonance mode was also the strongest that was calculated in the VS for each case. Furthermore, regarding these highest values of the resonance frequencies, it can be also observed that the value of the resonance frequency is strongly correlated with the chalcogenide composite. For the case of sulfur, which is the one with the lowest atomic mass, the highest value of resonance frequency was calculated, whereas, for tellurium, which had the highest atomic mass, the calculation led to the lowest value of the resonance frequency. Another common feature of this group of NPs was that for every examined case of chalcogenides, the strongest resonance was found for the one-dimensional case where x=16. As already described for ${Mg}_{16}S_{16}-1D,$ the strongest resonance was found at 431 ${cm}^{-1}$ with DOS value 2930 km/mol, for ${Mg}_{16}{Se}_{16}-1D$ at 338 ${cm}^{-1}$ with 1872 km/mol, and for ${Mg}_{16}{Te}_{16}-1D$ at 281 ${cm}^{-1}$ with DOS value 1590 km/mol. Furthermore, another common for all cases where x≤24 is related to the second strongest resonance. For all these cases, it was found that the second strongest resonance was calculated for x=24, as can be seen by the orange line in the relevant figures. It can be also seen by the relevant plots that DOS values for the calculated frequency resonances tend to have larger values for the case of sulfur. In contradiction, in the case of tellurium, the DOS values are the lowest calculated.

The examined group of ${Mg}_{32}S_{32}$ NPs also provided a plurality of both qualitative and quantitative observations, which helped to obtain the physical content of the vibrational properties of the examined nanostructures. For this group of nanostructures, although vibrational modes were calculated from very low frequencies, near 120 ${cm}^{-1}$, there is a frequency zone between 300 and 450 ${cm}^{-1}$ where strong resonances dominate, as can be seen by the relevant plot in Figure 2a. Among these resonances, the strongest is again for the one-dimensional case, ${Mg}_{32}S_{32}-1D$, which was calculated at 408 ${cm}^{-1}$ with 6874 km/mol. Another interesting point regarding ${Mg}_{32}S_{32}$ involves the exotic case, which is presented in Figure 2d. As can be seen from the relevant plot of Figure 2d, within the frequency zone between 300 and 450 ${cm}^{-1}$, the resonances for this case, which has a hole built within the nanostructure by proper extraction of atoms from its center, cause a slight shift in the resonances to higher frequencies when compared to the frequency modes of the simple ${Mg}_{32}S_{32}$, as shown in Figure 2b.

For a better view and deeper understanding of the resonance modes that were calculated in the framework of this research, a set of files where an animation of selected resonances is depicted. These files can be found in the Supplementary Materials of this work. For a more concise presentation, the animations were created from the group of calculations that refer to ${Mg}_{x}S_{x}$. As already mentioned, common features between ${Mg}_{x}Y_{x}$ NPs, which were analyzed for cases where x≤24, allow for this selection without loss of generality of the analysis. In each case of animated resonance, the name of the file indicates the selected case of the nanoparticle together with the represented frequency. In the animated files, as in the figures presented in this work, green spheres represent magnesium atoms and yellow spheres represent sulfur atoms. The characteristics of the two modes that are visualized for the ${Mg}_{4}S_{4}$ nanostructures can be easily recognized. For these two modes, the case of 301 ${cm}^{-1}$ shows a vertical vibration of the sulfur atoms, whereas for the mode with frequency 409 ${cm}^{-1}$, the relevant displacement has a longitudinal vibration. The strong vibration mode at 446 ${cm}^{-1}$ for the ${Mg}_{8}S_{8}$ nanostructures also provided a longitudinal vibration as the atoms oscillate parallel to the axis of the elongation of the nanoparticle. The same characteristics can be seen in the case of one-dimensional, ${Mg}_{16}S_{16}-1D$, where the strongest resonance that was calculated at 430 ${cm}^{-1}$ also has a longitudinal character. For both one-dimensional cases, for x=8 and for x=16, as can be clearly observed from the relevant animations, the strongest resonance is achieved when the magnesium atoms oscillate with a phase difference π compared to the relevant oscillations of the sulfur atoms. Evidently, the same behavior is also observed for the relevant one-dimensional case of ${Mg}_{32}S_{32}-1D$ and the frequency resonance at 410 ${cm}^{-1}$. Another interesting point that can be extracted from the animations regards ${Mg}_{32}S_{32}$ and the relevant case with the hole in the nanostructure, ${Mg}_{32}S_{32}-2DH$. For the latter, the strong resonance that was calculated at 442 ${cm}^{-1}$ should be attributed to the strong vibrations of the atoms at the periphery of the hole when compared to the rest vibrations of the atoms of the nanostructure. A close look at the relevant animation file will enlighten this behavior and the localization of the vibrational mode at the periphery of the hole. In more detail, magnesium atoms at the periphery of the hole oscillate in phase, whereas sulfur atoms oscillate with a phase difference π, thus resulting in oscillating in opposite directions.

## 5. Conclusions

This work aimed to provide numerical evidence regarding the vibrational spectrum of magnesium monochalcogenides nanoparticles. The examined magnesium monochalcogenide nanostructures were composed of a cubic unit of the form ${Mg}_{4}Y_{4}$, where Y=S,Se,Te, by properly elongating this unit along one, two, and three vertical directions. Within this research, an additional case was studied of an exotic nanoparticle that was composed of a hole in its center. Density functional theory was used for calculating the vibrational modes of the examined nanostructures after their geometry optimization. The structures studied, according to the results of the calculations, provided good structural stability, and the analysis of the results has led to a deeper understanding of their vibrational properties. A group of calculations was initially performed for the cases ${Mg}_{x}Y_{x}$, where x≤24. This group of calculations has led to the extraction of conclusions regarding some common properties between these nanostructures. For all these cases, the strongest vibrational modes were calculated for the cases where the elongation was along one direction. Thus, ${Mg}_{8}Y_{8}$, and ${Mg}_{16}Y_{16}-1D$ had the strongest calculated resonances among the examined cases. The frequencies of the vibrational spectrum of each one of these nanostructures were strongly related to the selection of the chalcogenide composite, Y, of the nanoparticle. For the case of sulfur, which was the one with the lowest atomic mass, the vibrational spectrum was found at a higher frequency area compared to tellurium, which had the highest atomic mass, and its vibrational spectrum was found at a lower frequency area. The study was continued by the calculation of the vibration spectrum of different cases of ${Mg}_{32}S_{32}$ nanoparticles. For this set of calculations, as in all previously examined nanostructures, the one-dimensional case provided the strongest resonance mode. It was also found that the creation of a hole in this nanoparticle led to a localization of the vibrations to the atoms on the periphery of the created hole, thus leading to another strong frequency mode.

This work aimed to investigate numerically the vibrational modes of a wide group of abundant, easy-to-find, and non-toxic nanoparticles. This research led to the finding of a group of common characteristics and differences of these nanostructures regarding their vibration modes, which could be very useful, especially to experimentalists. These findings could stand as a powerful tool regarding the response of the examined NPs when interacting with electrons, magnons, or photons.

## Data Availability

The raw data supporting the conclusions of this article will be made available by the authors on request.

## Supplementary Materials

The following supporting information can be downloaded at: https://www.mdpi.com/article/10.3390/nano14231918/s1. Video S1: Mg4S4_301.gif; Video S2: Mg4S4_409.gif; Video S3: Mg8S8_446.gif; Video S4: Mg16S16_1D_430.gif; Video S5: Mg16S16_416.gif; Video S6: Mg32S32_1D_410.gif; Video S7: Mg32S32_2DH_442.gif; Video S8: Mg32S32_414.gif; Table S1: Supplementary_all peaks_Mg24Y24 (Y=S,Se,Te).txt
