# Supplementary figures and images for: Vibrational Spectrum of Magnesium Monochalcogenide Nanoparticles
Source: Nanomaterials (Basel). 2024 Nov 28;14(23):1918. doi: 10.3390/nano14231918 (PMC11643477; doi:10.3390/nano14231918)

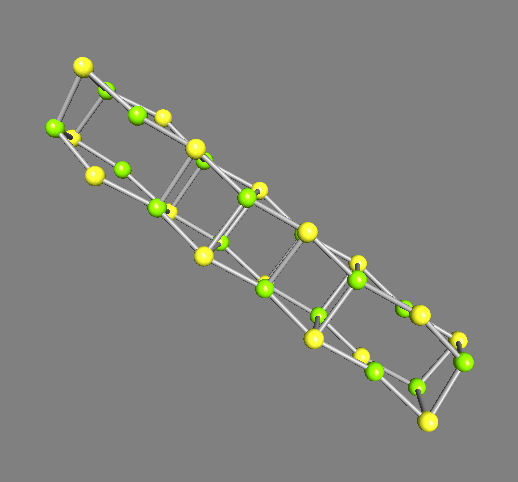

Supplement: Supplementary file 1 [file nanomaterials-14-01918-s001.zip › Mg16S16_1D_430.gif]

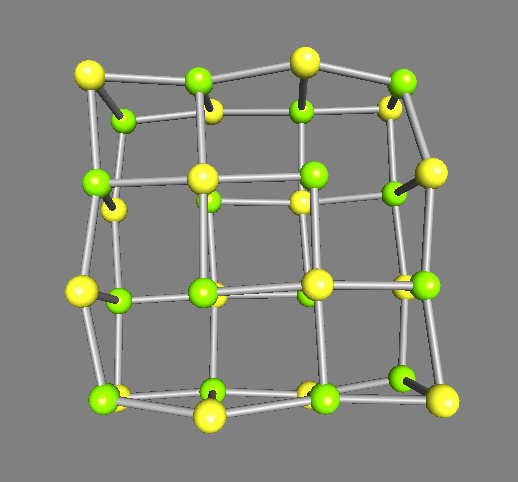

Supplement: Supplementary file 1 [file nanomaterials-14-01918-s001.zip › Mg16S16_416.gif]

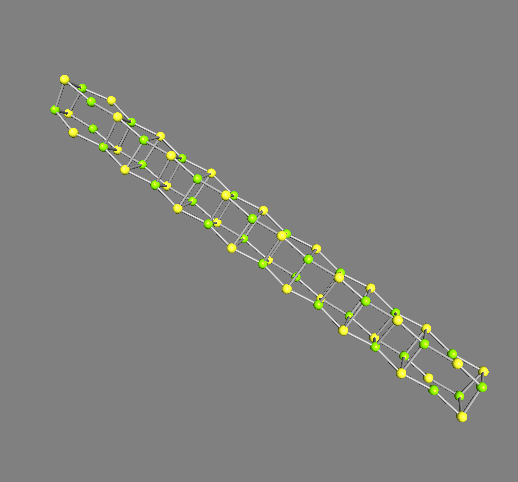

Supplement: Supplementary file 1 [file nanomaterials-14-01918-s001.zip › Mg32S32_1D_410.gif]

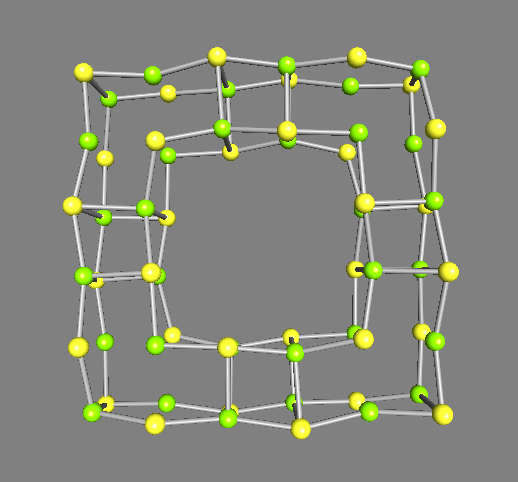

Supplement: Supplementary file 1 [file nanomaterials-14-01918-s001.zip › Mg32S32_2DH_442.gif]

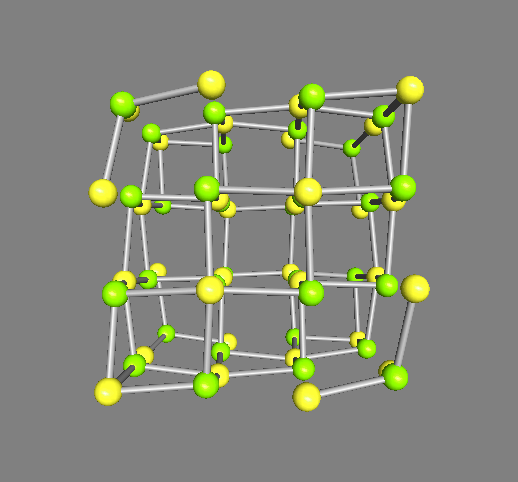

Supplement: Supplementary file 1 [file nanomaterials-14-01918-s001.zip › Mg32S32_414.gif]

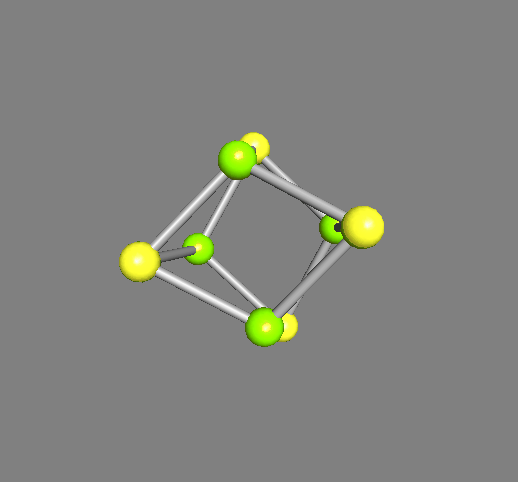

Supplement: Supplementary file 1 [file nanomaterials-14-01918-s001.zip › Mg4S4_301.gif]

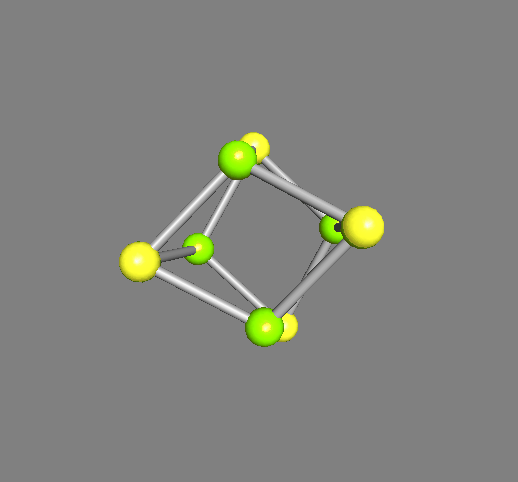

Supplement: Supplementary file 1 [file nanomaterials-14-01918-s001.zip › Mg4S4_409.gif]

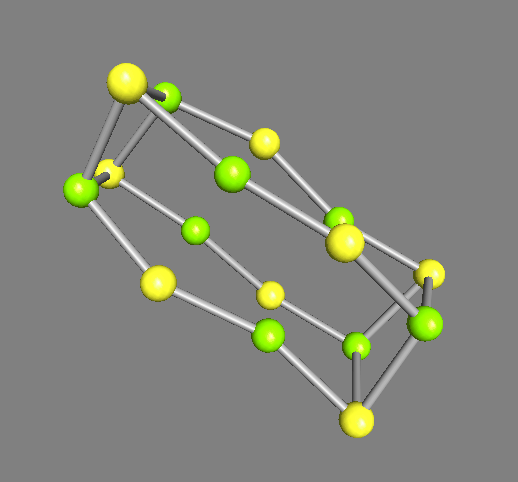

Supplement: Supplementary file 1 [file nanomaterials-14-01918-s001.zip › Mg8S8_446.gif]
